# Peripheral Erythrocytes Decrease upon Specific Respiratory Challenge with Grass Pollen Allergen in Sensitized Mice and in Human Subjects

**DOI:** 10.1371/journal.pone.0086701

**Published:** 2014-01-22

**Authors:** Galateja Jordakieva, Julia Wallmann, René Schmutz, Patrick Lemell, Michael Wegmann, Thomas Nittke, Martina Mittlböck, Heinz Fehrenbach, Jasminka Godnic-Cvar, René Zieglmayer, Erika Jensen-Jarolim

**Affiliations:** 1 Comparative Medicine, Messerli Research Institute of the University of Veterinary Medicine Vienna, Medical University of Vienna & University of Vienna, Vienna, Austria; 2 Institute of Occupational Medicine, Department of Internal Medicine II, Medical University of Vienna, Vienna, Austria; 3 Vienna Challenge Chamber (VCC), Vienna, Austria; 4 Priority Area Asthma & Allergy, Leibniz Center for Medicine and Biosciences, Research Center Borstel, Airway Research Center North, Member of the German Center for Lung Research, Borstel, Germany; 5 Section for Clinical Biometrics, Center for Medical Statistics, Informatics, and Intelligent Systems, Medical University of Vienna, Vienna, Austria; 6 Institute of Pathophysiology and Allergy Research, Center of Pathophysiology Infectiology and Immunology, Medical University of Vienna, Vienna, Austria; French National Centre for Scientific Research, France

## Abstract

**Background and Aims:**

Specific hyper-responsiveness towards an allergen and non-specific airway hyperreactivity both impair quality of life in patients with respiratory allergic diseases. We aimed to investigate cellular responses following specific and non-specific airway challenges locally and systemically in i) sensitized BALB/c mice challenged with grass pollen allergen Phl p 5, and in ii) grass pollen sensitized allergic rhinitis subjects undergoing specific airway challenge in the Vienna Challenge Chamber (VCC).

**Methods and Results:**

BALB/c mice (n = 20) were intraperitoneally immunized with grass pollen allergen Phl p 5 and afterwards aerosol challenged with either the specific allergen Phl p 5 (n = 10) or the non-specific antigen ovalbumin (OVA) (n = 10). A protocol for inducing allergic asthma as well as allergic rhinitis, according to the united airway concept, was used. Both groups of exposed mice showed significantly reduced physical activity after airway challenge. Specific airway challenge further resulted in goblet cell hyperplasia, enhanced mucous secretion, intrapulmonary leukocyte infiltration and lymphoid follicle formation, associated with significant expression of IL-4, IL-5 and IL-13 in splenocytes and also partially in lung tissue. Concerning circulating blood cell dynamics, we observed a significant drop of erythrocyte counts, hemoglobin and hematocrit levels in both mouse groups, challenged with allergen or OVA. A significant decrease in circulating erythrocytes and hematocrit levels after airway challenges with grass pollen allergen was also found in grass pollen sensitized human rhinitis subjects (n = 42) at the VCC. The effects on peripheral leukocyte counts in mice and humans however were opposed, possibly due to the different primary inflammation sites.

**Conclusion:**

Our data revealed that, besides significant leukocyte dynamics, particularly erythrocytes are involved in acute hypersensitivity reactions to respiratory allergens. A rapid recruitment of erythrocytes to the lungs to compensate for hypoxia is a possible explanation for these findings.

## Introduction

The prevalence of allergy mediated medical conditions is increasing worldwide and currently affecting approximately a fifth of the world population. [1] Especially allergic airway diseases are associated with significant quality of life limitations in affected patients. [2] Although less limiting than bronchial asthma, allergic rhinitis has been described as a global health concern causing major illness and disability worldwide. [3] Allergic rhinitis and allergic asthma are often found in the same patients and considered manifestations of the same inflammatory disease emphasized in different parts of the airways [2], [3].

Allergic asthma is defined as a chronic inflammatory disorder of the lower airways and is characterized by airway inflammation, persistent airway hyperresponsiveness (AHR) and intermittent acute, reversible airways obstruction. [4] Typically, the IgE-mediated, immediate obstruction is followed by a late phase reaction triggered by the release of inflammatory mediators like leukotrienes and cytokines (i.e. IL-4) from cells recruited to the lungs. This results in respiratory and, consequently, physical deficits of affected patients for a considerable time after an asthma attack. Additionally, in the absence of allergen, even exposure to unrelated antigens may trigger shortness of breath and wheezing, again limiting lung function and thus, seriously affecting the patients’ quality of life. Pallor is an additional well known clinical sign of severe asthma and has recently been described to occur in 10% of emergency cases [5].

Although mouse models of respiratory allergies exhibit some limitations compared to the human disease, they have provided important information about the conditions necessary for allergen sensitization. The common basis for the induction of Th2-type airway inflammation in mice is systemic sensitization followed by aerosol or intranasal challenges with the allergen. [6] Using this protocol renders an phenotype in mice mirroring the united airway concept in human patients, characterized by eosinophilic nasal and bronchial inflammation, increased Th2-type cytokines and antibody levels, mucus hyper-secretion and airway hyper-responsiveness. [7] However, the expression of each of these parameters can vary within the disease, with other sensitization protocols used [8], [9].

With respect to the profound impairment of capability in allergic airway diseases, we aimed to analyze peripheral blood cell dynamics during specific and non-specific aerosol allergen challenge in this study. For this purpose, data from BALB/c mice sensitized to Phl p 5 followed by aerosol challenges with allergen or non-specific control antigen was compared to data from grass pollen sensitized human rhinitis subjects who underwent airway challenge with specific allergen in the Vienna Challenge Chamber.

## Methods

### Mouse Study

#### Immunization of mice

BALB/c mice (female; 6–10 weeks) were purchased from Charles River Laboratories (Sulzfeld, Germany GmbH) and treated according to European Community rules of animal care [10] with the permission of the Austrian Ministry of Science (BMWF-66.012/0015-CIGT/2007); study approval was granted by the Ethics Commission of the Medical University of Vienna. BALB/c mice (n = 20) were immunized intraperitoneally (i.p.) with rPhl p 5a (10 µ g/100 µ l PBS) on days 0 and 21. Ten days later, mice (n = 10) were aerosol challenged with nebulized rPhl p 5a or OVA (protein and plasmid kindly provided by Prof. Arnd Petersen, Research Institute Borstel, Germany) (0.25 mg rPhl p 5a/100 ml PBS/challenge or Ovalbumin from chicken egg white 1%/100 mL PBS/challenge) with an ultrasonic nebulizer (Kendall, Aerodyne X, MTE Medizintechnik, Oldendorf, Germany) for 60 min twice daily with a four hours interval, on two consecutive days (day 31 and 32). Control groups were sensitized either with rPhl p 5a (n = 5) or PBS (n = 5) on days 0 and 21 and aerosol challenged 10 days later with PBS. One mouse group (n = 5) remained naïve. Measurements were performed on day 31 before, immediately after (day 32) and 48 hours after aerosol challenges (day 34) (Fig. S2A).

#### Assessment of activity

Activity was evaluated using InfraRed Actimeter frames equipped with infrared beam and detector units (Bioseb, France). Activity measurements were performed on day 31 before aerosol challenges, on day 32 and on day 34 (Fig. S2A).

#### Arterial blood analysis and differential blood cell counts in mice

Arterial blood samples from the tails of mice were taken and measured by a radiometer ABL800 FLEX (Wiener Neudorf, Austria). Differential blood cell counts were performed simultaneously with an Abbott Cell Dyn CD 3500 CS Analyzer, Vienna, Austria.

Measurements on naïve mice were performed on day 0 and immediately after sensitized mice were aerosol challenged with either Phl p 5 or OVA on day 31. Beside red blood cells (RBC) and white blood cells (WBC), hemoglobin (HGB) and hematocrit (HCT) were assessed by differential blood cell counts.

#### Histopathology

On day 35, mice were anesthetized (200 µ l 2% Rompun (Bayer, Leverkusen, Germany) and 5 ml Ketanest S (5****mg/ml); 600 µ l per mouse)) before fixation procedures were initiated. Lungs were fixed by intratracheal instillation performed with 4% paraformaldehyde in 0.1 M Sörensen’s phosphate buffer (12.0 mM NaH2PO4, 69.0 mM Na2HPO4) (pH 7.4), as described. [11] Lungs were embedded into paraffin as described earlier. [12] Systematic uniform random samples (SURS) of lung tissue were analyzed according to standard methods. Paraffin sections of 2 µ m in thickness were stained with periodic-acid-Schiff (PAS) for airway epithelial mucus. The surface area of mucus-secreting goblet cells (*Sgc*) per total surface area of airway epithelial basal membrane (*Sep*) and the volume of PAS-stained epithelial mucus (*Vmuc*) per *Sep* were determined using a computer-assisted Olympus BX61 microscope equipped with the newCAST stereology tool box (Visiopharm, Hoersholm, Denmark) according to following formulas:
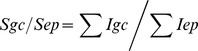


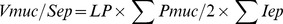




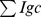
 = sum of intersections of test-lines with goblet cells


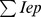
 = sum of all intersections of test-lines with epithelial basal membrane


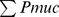
 = sum of all test-points hitting stored mucus

LP = test-line length per test-point at final magnification (490×).

The volume of lymphoid follicles (*Vfol*) per total surface area of airway epithelial basal membrane (*Sep*) was determined according to the formula:
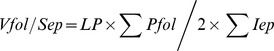




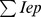
 = sum of all intersections of test-lines with epithelial basal membrane


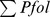
 = sum of all test-points hitting mucus

LP = test-line length per test-point at final magnification (490×).

#### Eosinophilic infiltration

Paraffin sections of 2 µ m in thickness were stained with Congo Red as previously reported [13] for the assessment of airway wall infiltration with eosinophilic granulocytes. The degree of infiltration within each field of view collected according to SURS, was ascribed to one of the three scores; (A) no (B) one or (C) two or more eosinophils in the airway wall.

#### RNA isolation and quantitative reverse transcriptase polymerase chain reaction (qRT-PCR)

Total RNA was extracted using the Trizol Reagent from Invitrogen (Invitrogen, Lofer, Austria) according to the manufacturer’s instructions. RNA was quantified by measuring the absorption at 260/280 nm using a NanoDrop spectrophotometer (Nanodrop Technologies Montchanin, DE, USA). 3 µ g of total RNA from each sample were reverse transcribed in a total volume of 20 µl using the high capacity cDNA reverse transcription kit (Applied Biosystems, Vienna, Austria), according to the manufacturer’s instructions. 2 µ l of cDNA were used for amplification by qRT-PCR which was performed with POWER SYBR®Green PCR Master Mix on an ABI StepOnePlus Realtime PCR System (Applied Biosystems, Vienna, Austria). IL-4, IL-5 and IL-13 mRNA levels were quantified by the comparative ΔΔCT method using the SDS software (Applied Biosystems, Foster City, CA, USA).

For each of the samples, relative expression was normalized to the housekeeping gene Hypoxanthine-phophoribosyl-transferase (HPRT). Pooled cDNA of naïve tissue was used as a calibrator and set the value 1; the relative expression levels of the other samples were compared hereto. Each sample was applied in duplicates. Following qRT-PCR program was used: 50°C for 2 min, 94°C for 2 min, followed by 40 cycles of 94°C for 15 s and 60°C for 30 s. Primers were designed using Primer Express (Applied Biosystems) and were located on different exons to prevent amplification of potentially contaminating genomic DNA.

Following primer sequences were used:

IL-4: fwd primer: ccatgcttgaagaagaactctagtgt

rev primer: attcatggtgcagcttatcgatg

IL-5: fwd primer: gaggcttcctgtccctatcataa

rev primer: tacccccacggacagtttga

IL-13: fwd primer: ccatctacaggacccagaggat

rev primer: gggaggctggagaccgtagt

### Human Study

#### Study subjects

Forty-two white subjects (n = 42, age 30±7.4, 88% male) (Tab. 1) with medical history of recurrent allergic airway symptoms were included. The subjects were divided in two groups differing in total duration of airway challenge and time points of baseline blood sampling. In *group 1* (n = 26) blood sampling was performed on the day before and immediately after allergen challenge in accordance with our mouse model. The total duration of continuous allergen exposure was 4 hours in this group. In *group 2* (n = 16) blood sampling was performed immediately before and after 2 hours of continuous allergen challenge. (Fig. S2B+C) Follow-up blood samples were collected approximately 1–3 weeks after allergen challenge to evaluate a return to baseline values.

**Table 1:** Demographic and clinical characteristics of study subjects.

	*Group 1*	*Group 2*	Total
**N**	26	16	**42**
**Sex (male)**	26 (100%)	11 (69%)	**37 (88%)**
**Age (years)**	30±7.5	29±7.5	**30±7.4**
**SPT**	10±2.6	9.6±2.6	**9.8±2.6**
**IgE RAST class**	4±1.2	4±1.2	**4±1.2**
**FEV1 baseline**	4.229±0.7	3.730±0.8	**4.039±0.8**
**FEV1 after challenge**	4.190±0.7	3.675±0.8	**3.994±0.7**

Data are mean±standard deviation, SPT = Skin-Prick-Test, RAST = Radioallergosorbent Test, FEV1 = forced expiratory volume in 1 second.

All subjects reported rhinitis symptoms (not attributed to infections and/or nasal abnormalities) manifesting for at least one year preceding the study; additionally few subjects reported occasional asthmatic symptoms. Sensitization to grass pollen allergen (Dac g, Phl p) was assessed by positive serologic and clinical examination (RAST [radioallergosorbent test] class 4±1.2, skin prick test 10±2.6****mm). Subjects had discontinued eventual immunomodulating medication prior to airway challenge (antihistamines: 72 hours; glucocorticosteroids: nasal 24 hours, bronchial 1 week, oral 12 weeks; other drugs potentially inducing enzymatic activity: 7 to 14 days). The study was approved by the ethics committee of the “Österreichische Arbeitsgemeinschaft für Klinische Pharmakologie” and performed between November 2010 and April 2011 after obtaining written informed consent of each subject.

#### Inclusion criteria

- Age 18–50 years.
- Healthy individuals (medical history, physical examination and routine laboratory without pathological findings), except for allergic rhinitis and intermittent or mild asthma [14] not requiring treatment and associated with normal baseline lung function (FEV1≥80%).
- Manifest allergic symptoms, positive skin prick test (≥4****mm) and elevated specific IgE (RAST class ≥2) results for grass pollen within the last 12 months preceding the study.

#### Exclusion criteria

- Planned or ongoing pregnancy during the study as well as breastfeeding.
- Nasal abnormalities (septum perforation, polyps, malformations, frequent epistaxis).
- Cold symptoms or acute infections of the upper respiratory tract within 3 weeks.
- Active respiratory tract disease, other than mild asthma not requiring treatment.
- Cigarette smoking within 6 months prior to study.
- Ongoing immunotherapy and/or immunomodulating medication.
- Known severe illness (e.g. heart disease) or malignancy.
- Alcohol and/or other drug abuse.
- Insufficient language abilities or compliance.
- Psychological disorders causing mental or legal incapacitation.

## Materials and Methods

A mixture of >93% pure pollen allergen (Allergon, Thermo Fisher Scientific) containing equal parts of pollen derived from Phleum pratense (phl p), Dactylis glomerata (Dac g), Lolium perenne (Lol p) and Anthoxanthum odoratum (Ant o) was used for airway challenge. The pollen were provided by the manufacturer with microbiological and chemical quality analysis certificates and dry stored at +2 to +10°C in their original containers. During airway challenge the pollen allergen mix was canalized into a feeding system (regulatory apparatus with vacuum outlet) connected to the air supply of the enclosed challenge chamber every five minutes. The allergen concentration in the challenge chamber was measured by persistent visual checking (Light Microscope, Olympus) of adhesive coated slides that were exposed to a constant air flow of 9–10 l/min (approximately equal to human nasal inspiratory flow) for five minutes inside a modified pollen trap (Burkard Co Ltd., Rickmansworth, England). The records were then saved to an Excel® spreadsheet every 5****min throughout the duration of a provocation session and used as basis for calculation of average, standard deviation, minimum and maximum value for the entire provocation session. Inside the challenge chamber climatic conditions were kept constant throughout each trial by a complex external air conditioning system combined with a processing plant for the supply and exhaustion of air. Temperature (24°C), humidity (40%), CO2 (%), air velocity (m/s) and air flow (m^3^/h or l/min) were monitored via several sensors (Alhborn) within the challenge chamber.

The subjects were exposed to the allergen for 4****h (*group 1*) or 2****h (*group 2*) in the provocation chamber. (Fig. S2B+C) Air concentration was kept at about ∼1500 pollen/m^3^ constantly (approximate air pollen concentration on a midsummer day). Every 15 min the study participants’ reported presence or absence of allergic symptoms, every 30 min the volume of air flow through the nose was measured by active anterior rhinomanometry, permitting indirect conclusions about the extent of swelling in the nasal mucosa and every 60 min spirometry was performed to assess changes in lung function (FEV1, FVC) for safety reasons.

### Differential Blood Cell Counts in Humans

Peripheral venous blood was sampled in ethylenediamine tetraacetic acid (EDTA) tubes at two time-points: on the day before (*group 1*) as in the mouse model or immediately before (*group 2*) and immediately after allergen challenge (both groups); a third blood sample (follow-up) was collected between 6 and 21 days after the allergen challenge sessions. EDTA blood samples were stored at +2°C to +10°C and analyzed within 2 days at an ISO 9001-certified laboratory (Labors Mühl-Speiser, Vienna, Austria) performing total and differential blood cell counts.

### Statistical Analysis

Continuous variables with a normal distribution are expressed as mean ± standard deviation (SD) or as median (minimum, maximum) if assumption of normal distribution was violated. Statistical comparisons between groups were made either by unpaired t-tests if data were normally distributed within groups or by Wilcoxon rank-sum test otherwise. Differences in before and after (paired) measurements are tested by paired t-test, if the differences were approximately normally distributed and by Wilcoxon’s signed rank test otherwise. All tests are based on a two-sided significance level of 0.05. The statistical software SPSS® (version 17.0 for Windows) was used for all statistical calculations.

## Results

### Mouse Model

#### Airway challenge with specific allergen and nonspecific antigen results in a significant reduction of motor activity in sensitized mice

**Figure 1:**
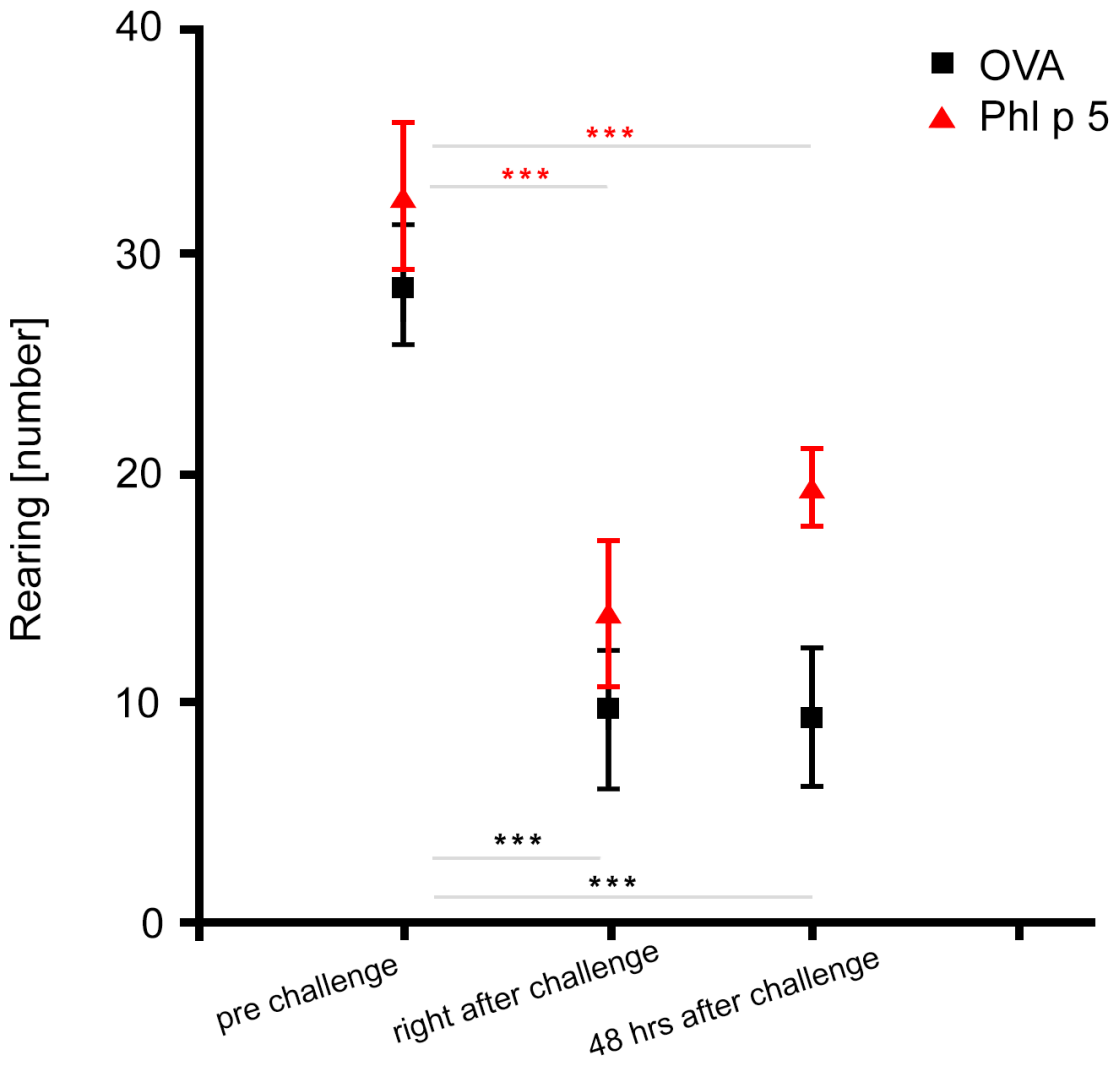
Physical activity after airway challenge. Rearing of Phl p 5-allergic in allergen (red squares) or nonspecific antigen (black points) challenged mice. Data are mean ± SD and n = 5 mice/group. ***, p<0.001.

The physical activity of mice was recorded before and after aerosolizations by counting the rearings (Z-axis movements) using an infrared beam at three different time-points; before, immediately after challenge and 48 hours later. Regardless of the allergen/antigen used for provocation of the airways, physical activity was drastically reduced in both mouse groups (Fig. 1), mirroring the clinical impairment in human patients. At the 48 hour time point, activity continued to be significantly reduced, irrespective of the agent used for challenge.

#### Peripheral erythrocyte parameters and leucocyte numbers after specific allergen aerosol challenges in sensitized mice

**Figure 2:**
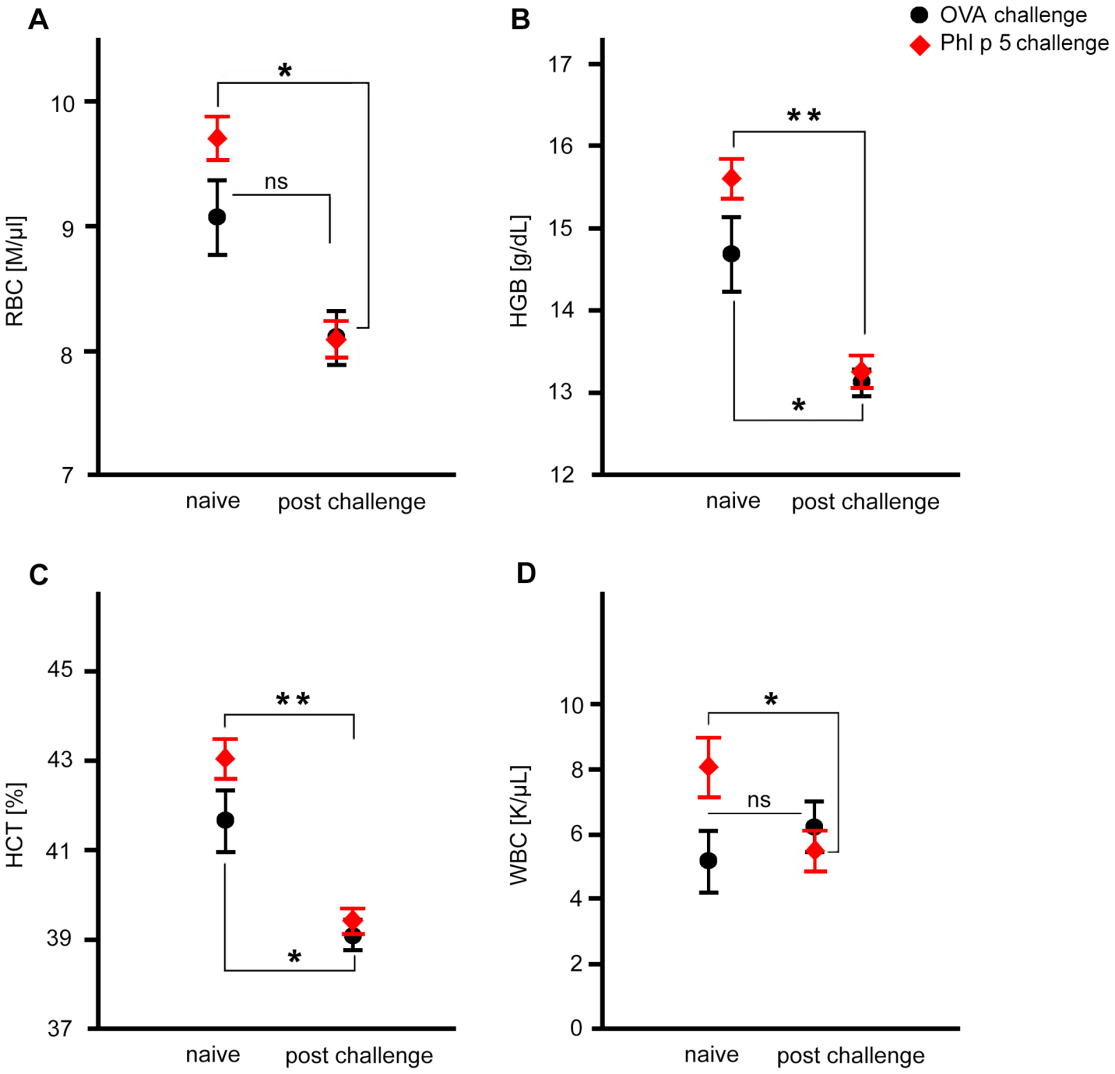
Specific and nonspecific airway provocation of allergic mice: impact on blood cells. (**A**) RBC, (**B**) HGB and (**C**) HCT levels of naïve, non-sensitized versus sensitized and Phl p 5 nebulized (red squares) and OVA challenged mice (black points) after airway challenge. Data are mean ± SD and n = 5 mice/group. *, p<0.05, **, p<0.01 and n.s. means not significant. (**D**) White blood cell counts (WBC) from naïve versus sensitized and Phl p 5 challenged (red squares) or OVA aerosolized mice (black points).

After aerosol challenge we observed significant decreases of peripheral red blood cell counts (RBC), hemoglobin (HGB) and hematocrit (HCT) compared to baseline values, suggestive of increased redistribution and dynamics of erythrocytes upon challenge with a specific allergen, and less with an irrelevant antigen (Fig. 2, A–C).

White blood cell counts (WBC) decreased in arterial blood of mice hypersensitive to Phl p 5, in the allergen-aerosol exposed group but remained almost unchanged in the group nebulized with control antigen OVA (Fig. 2D).

#### Allergen-specific, but less non-specific respiratory challenge, is associated with lymphoid airway inflammation

**Figure 3:**
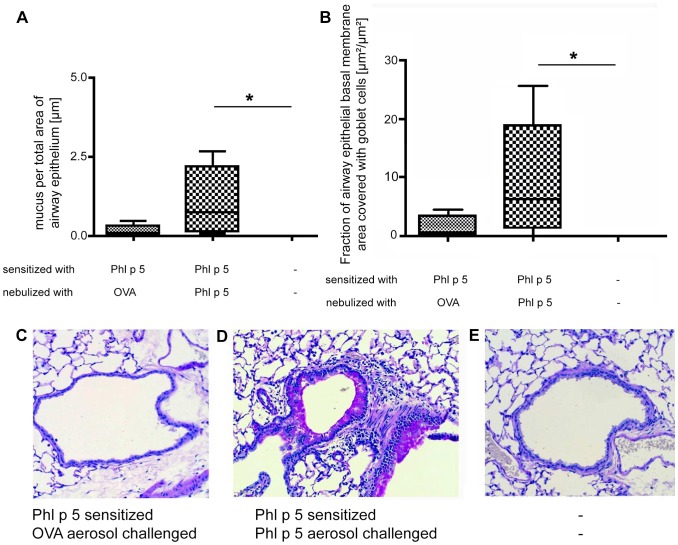
Goblet cell metaplasia, mucus production, lymphoid follicles and infiltration of airways with eosinophils after aerosol challenge. (**A**) Volume of epithelial mucus, (**B**) fraction of epithelial basal membrane covered by goblet cells. Representative lung histology from (**C**) Phl p 5 sensitized and OVA challenged (**D**), Phl p 5 sensitized and Phl p 5 challenged (**E**) naïve mice. Data are presented by boxplots (median, quartiles, minimum and maximum) and n = 5 mice/group. *, p<0.05.

**Figure 4:**
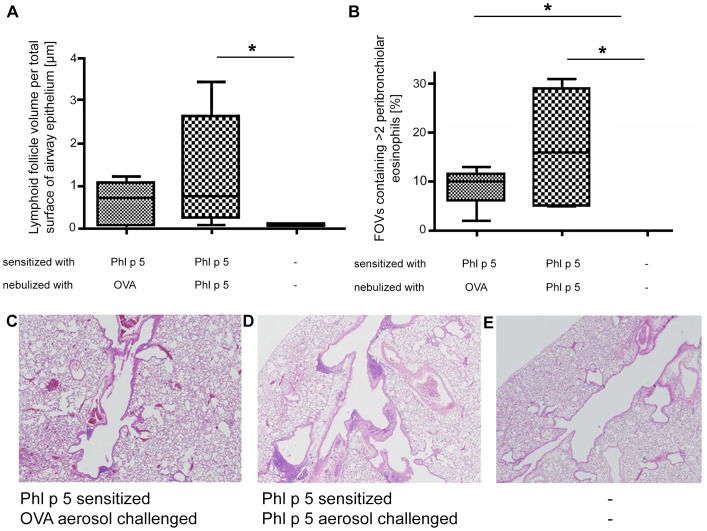
Aerosol allergen challenge specifically attracts leukocytes to lungs. (**A**) Volume of lymphoid follicles V/S (fol, epi) per total surface area of airway epithelial basal membrane [µm] (**B**) and percentage of airway walls containing eosinophils in fields of view (FOVs). Lung histology representatives with respect to lymphoid follicles from Phl p 5 sensitized and (**C**) OVA challenged or (**D**) Phl p 5 challenged and (**E**) naïve mice. Data are presented by boxplots (median, quartiles, minimum and maximum) and n = 5 mice/group. *, p<0.05, **, p<0.01 and n.s. means not significant.

Systematic uniform random samples (SURS) were collected from the entire lung and analyzed for goblet cell metaplasia, mucus production, lymphoid follicles and infiltration of the airways with eosinophils 64 hours after aerosol challenge (Fig. 3 and 4). In contrast to mice challenged with the allergen Phl p 5, mice immunized with Phl p 5 but challenged with OVA hardly exhibited any goblet cell metaplasia (Fig. 3A) or mucus production (Fig. 3B and C–E). Moreover, total volume of lymphoid follicles found peribronchially together with qualitative microscopic observations indicated that more and larger lymphoid follicles were present after mice were challenged with the specific allergen compared to OVA (Fig. 4A and C–E). Peripheral counts of lymphocytes were not affected (data not shown). Determining the percentage of airway walls containing eosinophils in the fields of view (FOVs) showed that eosinophilic infiltration was significantly increased in both, OVA and Phl p 5 challenged allergic mouse groups compared to naïve controls (Fig. 4B).

#### Phlp 5 sensitized mice aerosolized with the specific allergen exhibited higher Th2 cytokine mRNA production in lung tissue and splenocytes

As a measure for systemic immune deviation, expression of Th2 associated cytokines of spleen cells was investigated and compared to local effects in lungs. The spleens and the left lung of each mouse were isolated 64 hours after the last treatment. Relative expression of IL-4, IL-5 and IL-13 mRNA levels was quantified by qRT-PCR.

Aerosol challenges of Phl p 5-sensitized mice with OVA did neither elevate Th2 cytokine mRNA in spleens nor in lungs, as compared to naïve animals. As expected, challenges with the specific allergen Phl p 5 induced Th2 cytokine mRNA production in lungs and even more significantly in spleens (Fig. S1). These data indicate that significant transcription of IL-4, IL-5 and IL-13 is initiated as a consequence of specific allergen challenge and that it is a systemic event.

### Human Subjects

#### Peripheral erythrocyte numbers and hemoglobin decrease after specific allergen challenge in grass-pollen sensitized rhinitis subjects

Analogous to our findings in mice, the overall RBC counts were significantly decreased in sensitized subjects immediately after allergen airway challenge compared to baseline values (p<0.01); similarly HGB levels were reduced (p<0.05). These findings were significant in subjects exposed to grass pollen allergens for 4****h (*group 1*), however were not found in the smaller subject group exposed to allergen for 2****h only (*group 2*). The changes in HCT, MCV (mean corpuscular volume), MCH (mean corpuscular hemoglobin) or MCHC (mean corpuscular hemoglobin concentration) after allergen challenge did not reach statistical significance in any group (Tab. 2). In the follow-up blood samples no significant differences in RBC count, HGB or HCT were noted when compared to the respective baseline values before allergen challenge.

**Table 2:** Changes in number of peripheral blood cells before and after airway challenge with grass-pollen mix in subjects with allergic rhinitis, after 4****h (*group 1*) or 2****h (*group 2*) of allergen exposure; follow-up data shows the respective blood cell counts 6–21 days after airway allergen challenge.

	All subjects (n = 42)			Subgroups			
				*Group 1* (n = 26)		*Group 2* (n = 16)	
*Allergen challenge status*	*baseline*	*after*	*follow-up*	*baseline*	*after*	*baseline*	*after*
Red blood cells(RBC)×*10^12^/l*	5 (4.1, 5.7)	4.95 (4.1, 5.7)**	*5,01 (4.2, 5.6)*	5.08 (4.54, 5.57)	5 (4.46, 5.4)**	4.8 (4.1, 5.7)	4.85 (4.1, 5.7)
HGB *g/dl*	15 (13.2, 16.4)	14.7 (13.3, 16.4)*	*15 (12.8, 16.7)*	15.1(14.0, 16.1)	14.65 (13.7, 16.4)**	14.65 (13.2, 16.4)	14.7 (13.3, 16)
							
HCT *%*	44 (39, 49)	44 (39, 49)	*44 (39, 49)*	45 (42, 49)	43 (41, 49)	44 (39, 49)	44 (39, 48)
							
MCV *femtoliters/cell*	89 (82, 95)	89.5 (82, 98)	*89 (82, 98)*	89 (82, 95)	90 (82, 95)	88.5 (82, 95)	88.5 (82, 98)
							
MCH *picograms/cell*	30 (27, 33)	30 (27, 33)	30 (27, 33)*^a^	30 (27, 32)	30 (27, 32)	30 (27, 33)	29.5 (27, 33)
							
MCHC *g/dl*	34 (32, 36)	33 (32, 35)	*34 (32, 35)*	34 (32, 35)	33 (32, 35)	34 (32, 36)	34 (32, 35)
Leukocytes(WBC)×*10^9^/l*	6.4 (3.9, 10.4)	7.4 (4.3, 11.4)***	*6.33 (2.7, 12.7)*	6.74 (3.91, 9.26)	7.8 (4.49, 10.89)***	5.45 (3.9, 10.4)	6.4 (4.3, 11.4)**
Segmentedneutrophils×*10^9^/l*	3.05 (1.95, 7.1)	4.37 (1.9, 7.69)***	*3.29 (0.9, 8.9)*	3.12 (1.95, 5.88)	4.58 (1.92, 7.69)***	2.7 (2, 7.1)	3.4 (1.9, 7.1)**
Bandneutrophils×*10^9^/l*	0.1 (0, 0.3)	0.1 (0, 0.3)	*0.1 (0, 0.3)*	0.1 (0.1, 0.3)	0.1 (0.1, 0.2)*	0.1 (0, 0.2)	0.1 (0, 0.3)
Eosinophils×*10^9^/l*	0.22 (0.08, 0.65)	0.2 (0.05, 0.6)	*0.27 (0.08, 0.66)*	0.23 (0.1, 0.53)	0.19 (0.05, 0.5)	0.21 (0.08, 0.65)	0.25 (0.1, 0.6)
							
Basophils×*10^9^/l*	0.04 (0.01, 0.15)	0.06 (0.02, 0.12)*	*0.06 (0.02, 0.13)*	0.05 (0.03, 0.15)	0.06 (0.02, 0.12)	0.04 (0.01, 0.07)	0.06 (0.02, 0.07)**
							
Monocytes×*10^9^/l*	0.32 (0.2, 0.66)	0.37 (0.1, 0.68)	*0.39 (0.19, 0.58)*	0.34 (0.20, 0.66)	0.38 (0.24, 0.68)	0.3 (0.2, 0.5)	0.3 (0.1, 0.5)
							
Lymphocytes×*10^9^/l*	2.06 (1.1, 3.58)	2.05 (1.2, 3.3)	*1.94 (0.91, 3.14)*	2.22 (1.22. 3.58)	1.98(1.31, 2.86)*	1.85 (1.1, 2.6)	2.15 (1.2, 3.3)*
Thrombocytes×*10^9^/l*	239 (153, 418)	246 (159, 452)**	*235 (170, 409)*	232.5 (153, 341)	239 (159, 346)	269 (160, 418)	275 (170, 452)**

Data are median (minimum, maximum); *p<0.05, **p<0.01, ***p<0.001 compared to baseline values;

a Significantly more intra-individual decreases than increases from baseline.

#### Segmented neutrophils increase after allergen challenge in sensitized human subjects

In contrast to the described outcomes in aerosol-challenged mice we found significantly elevated peripheral leukocyte numbers immediately after allergen challenge in the grass-pollen sensitized human subjects (p<0.001) independent of the allergen exposure duration (4****h or 2****h; in *group 1* or *group 2*) (Fig. S3A). As depicted in Fig. S3B the most outstandingly increased leukocyte subset was that of segmented neutrophils (p<0.001 in both groups). In subjects who underwent 4****h of continuous allergen challenge absolute basophil numbers (p<0.05) were also significantly increased compared to baseline (pre-challenge) values. Overall post-challenge eosinophil numbers did not significantly differ from baseline values; also no clear trend was found for lymphocyte dynamics in the subgroup analysis. (Tab. 2) In the follow-up blood samples no significant dynamics were found in the leukocyte subsets, when compared to baseline values before allergen challenge, except for a shift in relative basophil ratio (p<0.05, data not shown) without significant changes in total basophil numbers.

Thrombocyte numbers increased after allergen exposure in both groups; however a statistical significance was only found in subjects after 2****h of continuous allergen challenge (p<0.01). In the follow-up blood samples, thrombocyte counts did not differ significantly from the respective baseline values.

## Discussion

In the present study we evaluated the local and systemic effects of specific allergen and non-specific antigen challenges in sensitized mice compared to data gained from airway challenges in human allergic rhinitis subjects at the Vienna Challenge Chamber. After allergen-specific as well as non-specific airway challenge in our study motor activity in mice immediately decreased and remained low until more than 48 hours after challenge which corresponds to sustained physical impairment in asthmatic patients [15] and allergic rhinitis patients [16].

In mouse models the degree of overall airway inflammation, depends on the experimental protocol used. [6] Here [17] immunization and aerosol challenges with Phl p 5 as the specific allergen rendered modest induction of mucus production, lymphoid follicle formation and eosinophilic infiltration in the proximal parts of the airways. Nebulization with the irrelevant antigen rendered milder inflammation and smaller lymphoid infiltration.

The levels of different Th2-associated cytokines and mRNA increased upon allergen challenge (S1) from splenocytes and lung tissues indicating systemic and local immune bias. The Th2-type lung inflammation was less pronounced upon non-specific aerosol challenge. IL-4 and IL-13 not only play a pivotal role in the production of IgE antibodies, but also contribute to narrowing of the airways. IL-4 also causes epithelial cells to swell and increases the contractibility of airway smooth muscle cells, thereby playing an essential role in AHR. [18], [19] IL-13 supports goblet cell metaplasia and mucus production. [20] Counter-regulation of Th2 cytokines also has therapeutic implications in allergic rhinitis. [21] IL-5 is primarily engaged in the recruitment of eosinophils, which negatively correlate with nasal airflow in asthma and rhinitis patients. [7] In our study, eosinophilic infiltration of the airways occurred to a comparable extent in both, specifically and non-specifically challenged sensitized mouse groups.

In mice, we observed a substantial decrease of red blood cell count, hemoglobin and hematocrit levels immediately following specific airway allergen challenge, with twofold significance upon specific provocation with Phl p 5.

We addressed the relevance of these phenomena in human grass pollen-sensitized rhinitis subjects undergoing respiratory airway challenge in the Vienna Challenge Chamber. [22]–[24] Like in the mouse study a significant decrease in erythrocyte and hemoglobin levels in the peripheral blood of the subjects was found after inhalative allergen challenge over 4 hours. The drop in circulating erythrocytes may to some extent be attributed to minor interstitial bleedings previously described as micro-epistaxis; [25] however taking the analogous results from our mouse model into consideration, we assume a compensatory re-distribution of erythrocytes to the respective site of inflammation for oxygen loading. Since erythrocytes may induce hypoxic lung inflammation, [26] the changes in erythrocyte counts upon allergen-challenge may also indicate a participation of the lungs in early wheezing, compatible with the United Airways concept. [27] No reports on effects of allergic asthma or rhinitis on red blood cell counts have been made so far, except sporadic evidence suggesting that human asthmatics may experience a decrease of hematocrit during recovery from acute episodes. [28] Our findings that acute allergic airway episodes are associated with in a significant fall of peripheral erythrocyte counts explain the facial pallor of the patients [29] and may contribute to the perception of respiratory discomfort [30].

The drop of leukocytes upon allergen challenge in the mouse study is in accordance with a previous observation in anaphylaxis, [31] but opposed the results from the human allergic subjects in our study, where peripheral leukocyte counts significantly increased after allergen challenge. A subset analysis showed that human segmented neutrophils and to some extent basophils accounted for the total leukocyte elevation, without relevant changes in less matured neutrophils or eosinophils. Neutrophils are known to have a crucial function in the allergy-mediated inflammatory response, for instance by release of proteases, [19], [20] myeloperoxidase (MPO) or elastase. [32]–[35] Neutrophils were elevated in blood and sputum after specific bronchial challenge in asthmatics; recently a rise in leukocytes in the delayed asthmatic response was observed proposing the monitoring of peripheral blood cell counts as a useful supplementary parameter to bronchial allergen challenge. [36], [37] In intermittent allergic rhinitis neutrophils are elevated in nasal lavage fluid within an hour after local allergen exposure and correlate with rhinitis symptoms. [38] Although conducted in allergic rhinitis rather than in bronchial asthma subjects, we also report a significant elevation of absolute and relative peripheral neutrophil numbers after allergen challenge. This reflects a first-line innate immune system response and supports the assumption that allergic rhinitis, like asthma, is a systemic condition [3], [39].

Taken together, we report significant peripheral blood cell dynamics as a non-classical parameter in acute allergic airways disease; in line with published data we confirm that white blood cells counts, specifically segmented neutrophils, significantly rise upon specific respiratory allergen challenge in human allergic rhinitis subjects. However, we consider the decrease in erythrocytes upon allergen challenge in sensitized mice as well as in human subjects as the most significant and novel result of our study. This finding may explain the profound systemic impairment of patients upon to specific and non-specific antigen inhalation.

## Supporting Information

Figure S1 **Nebulization of allergic mice with the allergen increases local and systemic Th2 mRNA cytokine levels. (A)** IL-4, **(B)** IL-5 and **(C)** IL-13 mRNA levels from spleens (left) and lungs (right) of challenged mice. Naïve organs served as negative controls. Data are presented by boxplots (median, quartiles, minimum and maximum) and n = 5 mice/group. *, p<0.05.
(TIF)

Figure S2 **Schematic protocol flow chart for allergen challenge in grass pollen-sensitized allergic rhinitis subjects, compared to the mouse model (A), and blood sampling after 4 h (** ***group 1*** **) (B) or 2 h (** ***group 2*** **) (C) of continuous allergen exposure.**
(TIF)

Figure S3 **Changes in peripheral blood cell counts in subjects after allergen challenge. (A)** White blood cells count and especially **(B)** total segmented neutrophils are significantly elevated after airway challenge. Data are represented by boxplots (median and quartiles) and n = 42. ***, p<0.001 respectively.
(TIF)

## References

[1] Pawankar R, Bunnag C, Khaltaev N, Bousquet J (2012) Allergic Rhinitis and Its Impact on Asthma in Asia Pacific and the ARIA Update 2008. World Allergy Organ J 5: S212–217. 23268481 10.1097/WOX.0b013e318201d831 PMC3488935

[2] Bousquet J, Schunemann HJ, Samolinski B, Demoly P, Baena-Cagnani CE, et al (2012) Allergic Rhinitis and its Impact on Asthma (ARIA): achievements in 10 years and future needs. J Allergy Clin Immunol 130: 1049–1062. 23040884 10.1016/j.jaci.2012.07.053

[3] Bousquet J, Khaltaev N, Cruz AA, Denburg J, Fokkens WJ, et al (2008) Allergic Rhinitis and its Impact on Asthma (ARIA) 2008 update (in collaboration with the World Health Organization, GA(2)LEN and AllerGen). Allergy 63 Suppl 868–160. 18331513 10.1111/j.1398-9995.2007.01620.x

[4] Bousquet J, Jeffery PK, Busse WW, Johnson M, Vignola AM (2000) Asthma. From bronchoconstriction to airways inflammation and remodeling. Am J Respir Crit Care Med 161: 1720–1745. 10806180 10.1164/ajrccm.161.5.9903102

[5] Bazaraa HM, El Houchi S, Rady HI (2012) Profile of patients visiting the pediatric emergency service in an Egyptian university hospital. Pediatr Emerg Care 28: 148–152. 22270495 10.1097/PEC.0b013e3182442eeb

[6] McCusker CT (2004) Use of mouse models of allergic rhinitis to study the upper and lower airway link. Curr Opin Allergy Clin Immunol 4: 11–16. 15090913 10.1097/00130832-200402000-00004

[7] Yukselen A, Kendirli SG, Yilmaz M, Altintas DU, Karakoc GB (2012) Correlation between nasal eosinophils and nasal airflows in children with asthma and/or rhinitis monosensitised to house dust mites. Allergol Immunopathol (Madr). 10.1016/j.aller.2012.07.001 23122003

[8] Epstein MM (2006) Are mouse models of allergic asthma useful for testing novel therapeutics? Exp Toxicol Pathol 57 Suppl 241–44. 16580828 10.1016/j.etp.2006.02.005

[9] Wallmann J, Epstein MM, Singh P, Brunner R, Szalai K, et al (2010) Mimotope vaccination for therapy of allergic asthma: anti-inflammatory effects in a mouse model. Clin Exp Allergy 40: 650–658. 19958367 10.1111/j.1365-2222.2009.03392.x PMC2999747

[10] Waldegrave W (1986) Directive CEE. J Officiel Communautés. 1–28.

[11] Forkert PG (1995) CYP2E1 is preferentially expressed in Clara cells of murine lung: localization by in situ hybridization and immunohistochemical methods. Am J Respir Cell Mol Biol 12: 589–596. 7766423 10.1165/ajrcmb.12.6.7766423

[12] Wegmann M, Fehrenbach H, Fehrenbach A, Held T, Schramm C, et al (2005) Involvement of distal airways in a chronic model of experimental asthma. Clin Exp Allergy 35: 1263–1271. 16238784 10.1111/j.1365-2222.2005.02306.x

[13] Pali-Scholl I, Yildirim AO, Ackermann U, Knauer T, Becker C, et al (2008) Anti-acids lead to immunological and morphological changes in the intestine of BALB/c mice similar to human food allergy. Exp Toxicol Pathol 60: 337–345. 18524557 10.1016/j.etp.2008.03.004

[14] Global Initiative for Asthma (GINA) NH, Lung and Blood Institute (NHLBI) Bethesda (MD); (2006) Global strategy for asthma management and prevention.

[15] Everhart RS, Smyth JM, Santuzzi AM, Fiese BH (2010) Validation of the Asthma Quality of Life Questionnaire with momentary assessments of symptoms and functional limitations in patient daily life. Respir Care 55: 427–432. 20406510

[16] Canonica GW, Bousquet J, Mullol J, Scadding GK, Virchow JC (2007) A survey of the burden of allergic rhinitis in Europe. Allergy 62 Suppl 8517–25. 10.1111/j.1398-9995.2007.01549.x 17927674

[17] Mojtabavi N, Dekan G, Stingl G, Epstein MM (2002) Long-lived Th2 memory in experimental allergic asthma. J Immunol 169: 4788–4796. 12391188 10.4049/jimmunol.169.9.4788

[18] Corry DB, Folkesson HG, Warnock ML, Erle DJ, Matthay MA, et al (1996) Interleukin 4, but not interleukin 5 or eosinophils, is required in a murine model of acute airway hyperreactivity. J Exp Med 183: 109–117. 8551213 10.1084/jem.183.1.109 PMC2192426

[19] Galli SJ, Tsai M, Piliponsky AM (2008) The development of allergic inflammation. Nature 454: 445–454. 18650915 10.1038/nature07204 PMC3573758

[20] Mitchell J, Dimov V, Townley RG (2010) IL-13 and the IL-13 receptor as therapeutic targets for asthma and allergic disease. Curr Opin Investig Drugs 11: 527–534. 20419598

[21] Erin EM, Zacharasiewicz AS, Nicholson GC, Tan AJ, Higgins LA, et al (2005) Topical corticosteroid inhibits interleukin-4, -5 and -13 in nasal secretions following allergen challenge. Clin Exp Allergy 35: 1608–1614. 16393327 10.1111/j.1365-2222.2005.02381.x

[22] Zieglmayer PU (2013) Are results of environmental exposure units transferable to real-life exposure? Curr Opin Allergy Clin Immunol. 10.1097/ACI.0b013e328360c7b6 23549153

[23] Horak F, Zieglmayer P, Zieglmayer R, Lemell P, Devillier P, et al. (2009) Early onset of action of a 5-grass-pollen 300-IR sublingual immunotherapy tablet evaluated in an allergen challenge chamber. J Allergy Clin Immunol 124: 471–477, 477 e471. 10.1016/j.jaci.2009.06.006 19647862

[24] Horak F, Jager S, Berger U (1992) Onset and duration of the effects of three antihistamines in current use–astemizole, loratadine and terfenadine forte–studied during prolonged, controlled allergen challenges in volunteers. J Int Med Res 20: 422–434. 1451923 10.1177/030006059202000507

[25] Park YJ, Repka-Ramirez MS, Naranch K, Velarde A, Clauw D, et al (2002) Nasal lavage concentrations of free hemoglobin as a marker of microepistaxis during nasal provocation testing. Allergy 57: 329–335. 11906364 10.1034/j.1398-9995.2002.1o3253.x

[26] Kiefmann R, Rifkind JM, Nagababu E, Bhattacharya J (2008) Red blood cells induce hypoxic lung inflammation. Blood 111: 5205–5214. 18270324 10.1182/blood-2007-09-113902 PMC2384143

[27] Compalati E, Ridolo E, Passalacqua G, Braido F, Villa E, et al (2010) The link between allergic rhinitis and asthma: the united airways disease. Expert Rev Clin Immunol 6: 413–423. 20441427 10.1586/eci.10.15

[28] Gopalan AV, Govindan UT, Govindaraj M (1983) Asthma and chronic bronchitis: contrasting changes in body weight and hematocrit values during recovery from acute episodes. J Asthma 20: 53–55. 6853429 10.3109/02770908309070914

[29] Przybilla B, Ring J, Enders F, Winkelmann H (1991) Stigmata of atopic constitution in patients with atopic eczema or atopic respiratory disease. Acta Derm Venereol 71: 407–410. 1684469

[30] Scano G, Stendardi L (2006) Dyspnea and asthma. Curr Opin Pulm Med 12: 18–22. 16357574 10.1097/01.mcp.0000199003.46038.82

[31] Krishnamurthy D, Starkl P, Szalai K, Roth-Walter F, Diesner SC, et al (2012) Monitoring neutrophils and platelets during casein-induced anaphylaxis in an experimental BALB/c mouse model. Clin Exp Allergy 42: 1119–1128. 22702510 10.1111/j.1365-2222.2012.04012.x

[32] Monteseirin J (2009) Neutrophils and asthma. J Investig Allergol Clin Immunol 19: 340–354. 19862934

[33] Takeyama K, Agusti C, Ueki I, Lausier J, Cardell LO, et al (1998) Neutrophil-dependent goblet cell degranulation: role of membrane-bound elastase and adhesion molecules. Am J Physiol 275: L294–302. 9700090 10.1152/ajplung.1998.275.2.L294

[34] Nadel JA, Takeyama K, Agusti C (1999) Role of neutrophil elastase in hypersecretion in asthma. Eur Respir J 13: 190–196. 10836347 10.1034/j.1399-3003.1999.13a35.x

[35] Westin U, Lundberg E, Wihl JA, Ohlsson K (1999) The effect of immediate-hypersensitivity reactions on the level of SLPI, granulocyte elastase, alpha1-antitrypsin, and albumin in nasal secretions, by the method of unilateral antigen challenge. Allergy 54: 857–864. 10485390 10.1034/j.1398-9995.1999.00938.x

[36] Pelikan Z (2011) Delayed asthmatic response to bronchial challenge with allergen-mediators, eicosanoids, eosinophil and neutrophil constituents in the blood and urine. Respiration 82: 225–236. 21454958 10.1159/000324542

[37] Pelikan Z (2012) Cellular Profiles in Peripheral Blood Accompanying Particular Asthmatic Response Types. International Journal of Clinical Medicine 3: 485–497.

[38] Fransson M, Benson M, Wennergren G, Cardell LO (2004) A role for neutrophils in intermittent allergic rhinitis. Acta Otolaryngol 124: 616–620. 15267182 10.1080/00016480310015173

[39] Togias A (2004) Systemic effects of local allergic disease. J Allergy Clin Immunol 113: S8–14. 14694344 10.1016/j.jaci.2003.09.051

