# Supplementary figures and images for: Peripheral Erythrocytes Decrease upon Specific Respiratory Challenge with Grass Pollen Allergen in Sensitized Mice and in Human Subjects
Source: PLoS One. 2014 Jan 22;9(1):e86701. doi: 10.1371/journal.pone.0086701 (PMC3899302; doi:10.1371/journal.pone.0086701)

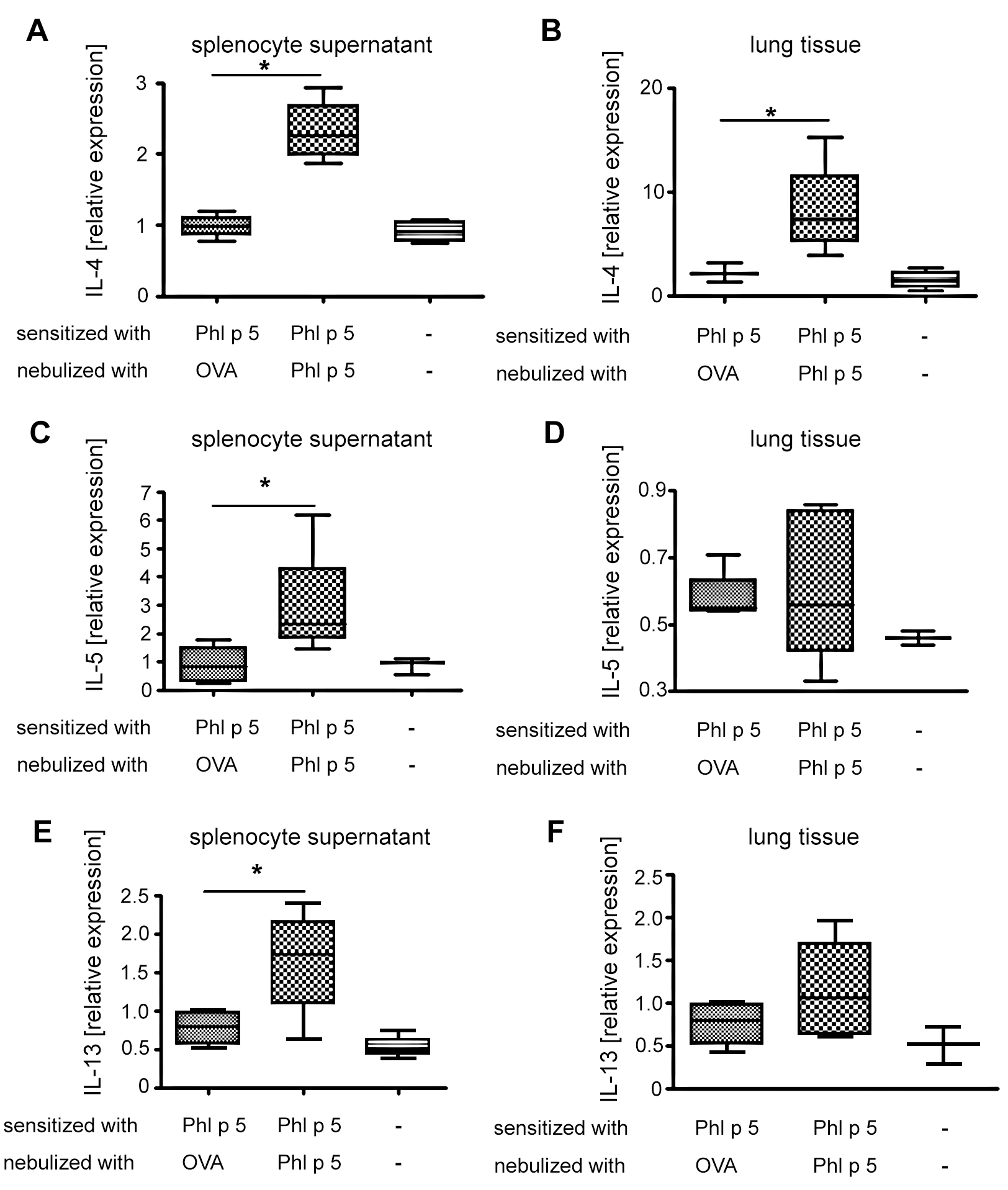

Supplement: Figure S1 — Nebulization of allergic mice with the allergen increases local and systemic Th2 mRNA cytokine levels. (A) IL-4, (B) IL-5 and (C) IL-13 mRNA levels from spleens (left) and lungs (right) of challenged mice. Naïve organs served as negative controls. Data are presented by boxplots (median, quartiles, minimum and maximum) and n = 5 mice/group. *, p<0.05. (TIF) [file pone.0086701.s001.tif]

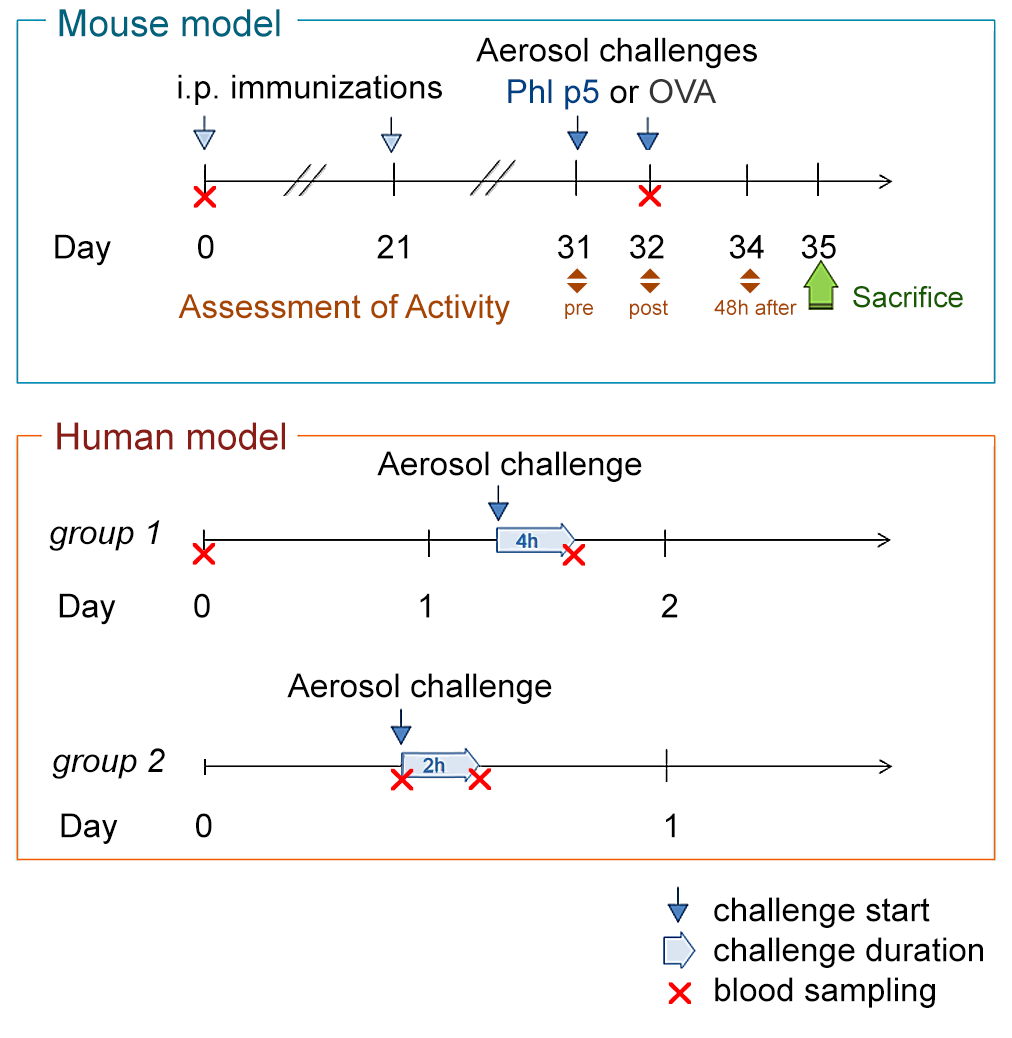

Supplement: Figure S2 — Schematic protocol flow chart for allergen challenge in grass pollen-sensitized allergic rhinitis subjects, compared to the mouse model (A), and blood sampling after 4 h ( group 1 ) (B) or 2 h ( group 2 ) (C) of continuous allergen exposure. (TIF) [file pone.0086701.s002.tif]

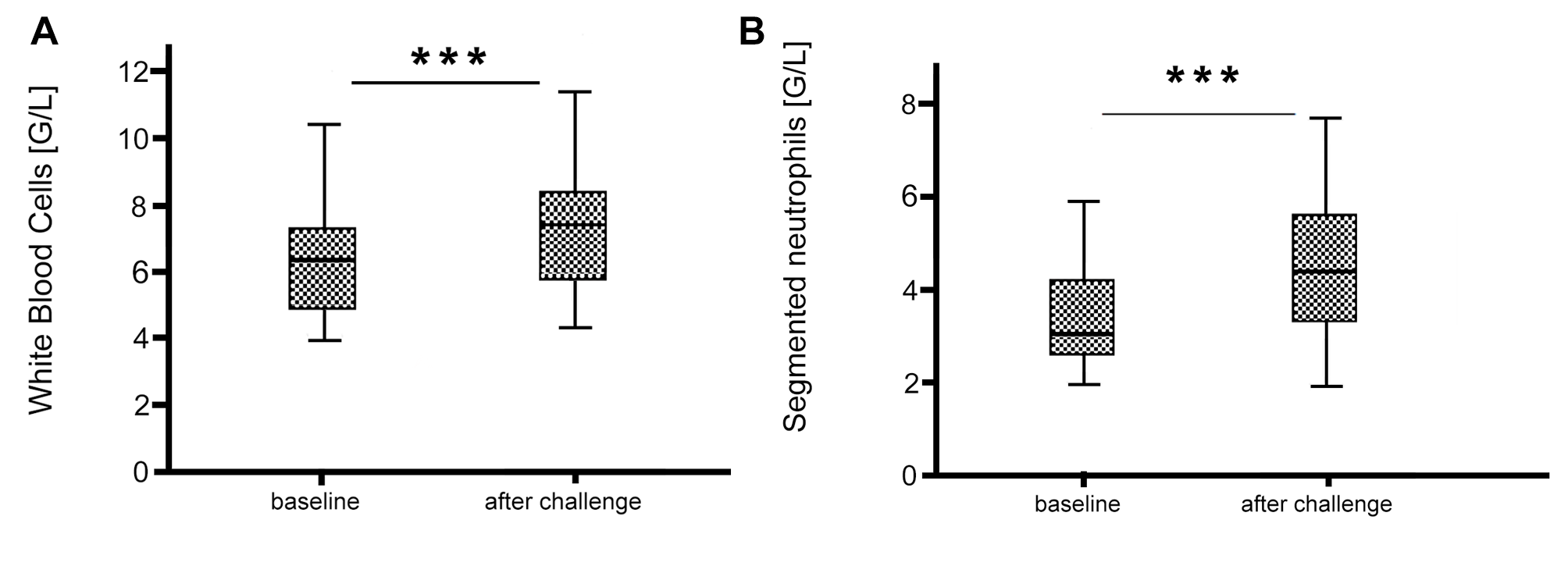

Supplement: Figure S3 — Changes in peripheral blood cell counts in subjects after allergen challenge. (A) White blood cells count and especially (B) total segmented neutrophils are significantly elevated after airway challenge. Data are represented by boxplots (median and quartiles) and n = 42. ***, p<0.001 respectively. (TIF) [file pone.0086701.s003.tif]
